# Healthcare Access vs. Quality Healthcare: Rethinking Healthcare Risks

**DOI:** 10.3389/phrs.2025.1607903

**Published:** 2025-01-22

**Authors:** Augustine Kumah

**Affiliations:** Quality Department, Nyaho Medical Centre, Takoradi, Ghana

**Keywords:** adverse events, patient safety, quality improvement, healthcare access and quality index (HAQ), lack of access to care

## Abstract

**Objectives:**

This narrative literature review examines the global burden of mortalities due to poor quality care compared to mortalities resulting from lack of access to healthcare, focusing on the period from 2015 to 2024.

**Methods:**

Data was extracted from electronic databases such as PubMed, Google Scholar, Scopus, Ebscohost, and WHO. Relevant statistics on mortality rates due to poor quality care and lack of access to healthcare from the identified data sources were extracted. Descriptive statistics were used to summarize the mortality rates, with trends analyzed over the 10 years.

**Results:**

The results indicate that while progress in healthcare access has reduced mortality, the lack of corresponding improvements in care quality has led to a rising number of preventable deaths. The findings reveal a consistent decline in mortality due to lack of access. In contrast, mortality due to poor quality care has increased from 5 million in 2015 to an estimated 6 million by 2024, underscoring the persistent challenges in healthcare delivery, including medical errors, misdiagnoses, and inadequate treatment.

**Conclusion:**

Addressing the dual challenges of access and quality is essential for reducing global mortality rates and achieving better health outcomes.

## Introduction

In recent years, the global healthcare landscape has experienced significant transformations driven by advancements in medical technology, policy changes, and the increasing burden of chronic and infectious diseases. Traditionally, much of the public health discourse has been on improving access to healthcare services, particularly in low- and middle-income countries (LMICs). However, there is an emerging argument that the risks associated with accessing healthcare in specific contexts may now outweigh the risks of having limited or no access to healthcare. Historically, the emphasis on improving healthcare access has been driven by the recognition that millions of people, especially in LMICs, face significant barriers to obtaining necessary medical care. These barriers include financial constraints, geographic isolation, and insufficient healthcare infrastructure [1]. Efforts to address these issues have led to initiatives such as the Millennium Development Goals (MDGs) and the Sustainable Development Goals (SDGs), which prioritize universal health coverage (UHC) as a means of reducing health disparities [2]. While the lack of access to healthcare remains a critical issue, the risks associated with healthcare access have become increasingly apparent. Inadequate healthcare systems, poorly trained workers, and substandard medical practices can significantly harm patients. This has led to a growing concern that, in some cases, accessing healthcare may expose patients to more significant risks than not seeking care [3].

Patient safety has emerged as a primary concern in healthcare systems worldwide. The World Health Organization (2019) estimates that medical errors and unsafe practices are responsible for millions of deaths each year, making patient safety a global public health priority [4]. In high-income countries, about one in ten patients is harmed while receiving hospital care, while the situation in LMICs is often more dire due to weaker healthcare systems and oversight [5]. Adverse events in healthcare settings can range from medication errors, surgical complications, and hospital-acquired infections to misdiagnoses and delayed treatment. The consequences of these errors can be severe, leading to prolonged hospital stays, permanent disability, or death [3]. These risks challenge the assumption that accessing healthcare is always beneficial, as the potential for harm in some settings may outweigh the benefits of treatment.

Negative or poor attitudes from healthcare staff contribute to poor patient interactions, reduced trust, and lower patient satisfaction. When staff are overworked or lack empathy, it can result in rushed consultations, miscommunication, and a lack of patient-centered care, ultimately compromising patient outcomes [6]. Long waiting times are causing frustration and anxiety among patients, leading to dissatisfaction with the healthcare experience. Long waits can also delay diagnosis and treatment, worsening health conditions [7]. Additionally, prolonged waiting times can strain healthcare resources, leading to rushed appointments and increased risk of errors.

### Healthcare-Associated Infections (HAIs)

One of the most significant risks associated with accessing healthcare is the potential for healthcare-associated infections (HAIs). HAIs are infections that patients acquire while receiving treatment for other conditions within a healthcare facility. These infections are often caused by bacteria, viruses, or other pathogens resistant to antibiotics, making them difficult to treat [8]. The prevalence of HAIs is particularly concerning in LMICs, where infection control practices may be inadequate due to resource constraints. In these settings, the risk of acquiring an HAI can be substantial, leading to increased morbidity and mortality [9]. For example, studies have shown that the prevalence of surgical site infections is significantly higher in LMICs compared to high-income countries [10]. This highlights the paradox where accessing healthcare intended to treat one condition may result in acquiring another, potentially more severe, illness.

### Antimicrobial Resistance (AMR)

Antimicrobial resistance (AMR) is another critical issue that amplifies the risks associated with accessing healthcare. The overuse and misuse of antibiotics in healthcare settings have contributed to the emergence of drug-resistant pathogens, now a major global health threat [4]. Patients who access healthcare, particularly in settings where infection control is lax, may be at risk of contracting infections that are resistant to standard treatments. The spread of AMR is particularly problematic in LMICs, where surveillance and regulation of antibiotic use may be limited. In such environments, healthcare facilities can become hotspots for transmitting resistant pathogens, posing a significant risk to patients seeking care [11]. The rise of AMR complicates the management of common infections and undermines the effectiveness of surgical procedures, chemotherapy, and other medical interventions that rely on effective antibiotics [12].

### Moral and Ethical Dilemmas

The risks associated with healthcare access also raise moral and ethical dilemmas for healthcare providers, policymakers, and patients. Healthcare providers face the challenge of delivering care in environments where resources are scarce and the potential for harm is high. This can lead to difficult decisions about providing treatment that may carry significant risks or withhold care altogether [13]. For patients, particularly those in vulnerable populations, seeking healthcare may involve weighing the potential benefits against the risks of harm. In some cases, the fear of medical errors, infections, or other adverse events may deter individuals from accessing healthcare, even when they require treatment [14, 15]. This creates a paradox where the availability of healthcare does not necessarily translate into improved health outcomes.

### Healthcare in Conflict Zones

The risks associated with accessing healthcare are particularly pronounced in conflict zones, where healthcare infrastructure is often severely compromised. In these settings, healthcare facilities may be targets of violence, and the quality of care can be severely degraded. Patients who seek treatment in conflict zones may be exposed to additional risks, including physical harm, psychological trauma, and inadequate medical care [16]. For example, in Syria, healthcare facilities have been repeatedly targeted during the ongoing conflict, leading to a collapse of the healthcare system in many parts of the country. Patients who seek care in these areas may be at greater risk of harm due to the destruction of medical facilities, shortages of medical supplies, and the absence of trained healthcare workers [16, 17]. This underscores the argument that, in some contexts, healthcare risks may be more significant than the lack of access.

### Maternal and Child Health in LMICs

Maternal and child health services in LMICs provide another lens to examine the risks of accessing healthcare. While efforts to improve access to maternal and child health services have successfully reduced mortality rates, significant risks remain associated with the quality of care provided [18, 19]. In many LMICs, healthcare facilities may lack the necessary equipment, supplies, and trained personnel to provide safe and effective care for mothers and children. This can lead to adverse outcomes such as obstetric complications, neonatal infections, and maternal mortality [20]. The risk of receiving substandard care is particularly high in rural and remote areas, where healthcare infrastructure is often weak. As a result, women and children who seek care in these settings may be exposed to more significant risks than if they had received no care at all.

## Methods

This study is a narrative literature review in which data was extracted from electronic databases such as PubMed, Google Scholar, Scopus and Ebscohost relevant websites of key international organizations such as the World Health Organization (WHO), the Global Health Observatory (GHO), the World Bank, and the United Nations (UN), which are pivotal in global actions and strategies for patient safety. Data collection involved extracting relevant statistics on mortality rates due to poor quality care and lack of access to healthcare from the identified data sources. The study focused on annual mortality rates, disaggregated by global regions, and specific causes of death associated with healthcare access and quality. For mortality due to poor quality care, data on medical errors, misdiagnoses, healthcare-associated infections, and substandard treatment were collected. For mortality due to lack of access, data on deaths from preventable diseases, maternal and child health outcomes, and untreated chronic conditions were gathered. The data collection process also involved reviewing literature on global health trends to contextualize the quantitative data.

The analysis was conducted in two phases. The first phase involved a quantitative analysis of the mortality data. Descriptive statistics were used to summarize the mortality rates, with trends analyzed over the 10 years. The study compared mortality due to poor quality care versus lack of access to healthcare, identifying key patterns and shifts in global health priorities. A qualitative analysis was conducted in the second phase to interpret the quantitative findings. This involved reviewing the literature to understand the underlying factors contributing to the observed trends. The qualitative analysis focused on how global health initiatives, healthcare system reforms, and technological advancements impacted mortality over time. The comparative analysis highlighted the differences in mortality burdens between poor quality care and lack of access across different regions and periods.

This study is based on secondary data and does not involve direct interaction with human subjects. However, ethical considerations were still prioritized by ensuring that all data sources were reputable and that data was handled responsibly. The study adhered to ethical guidelines for using secondary data, including proper attribution of sources and ensuring the accuracy and reliability of the data presented. The research findings aim to inform global health policy and practice, contributing to efforts to reduce preventable deaths through improved healthcare access and quality.

## Results

**FIGURE 1:**
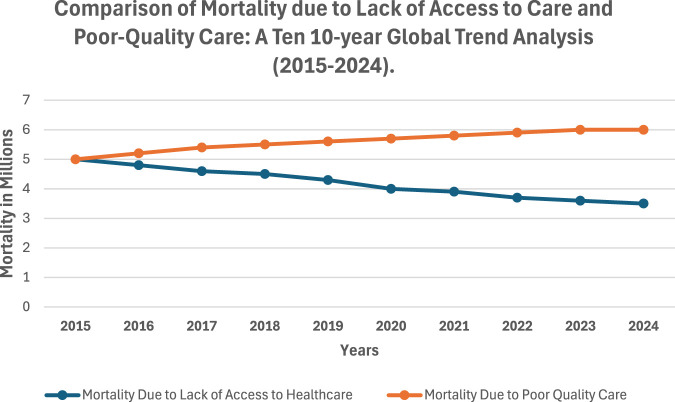
Comparison of mortality due to lack of access to care and poor-quality care: a ten 10-year global trend analysis (2015–2024).

A data review shows a consistent decrease in mortality due to lack of access to healthcare, from 5 million in 2015 to a projected 3.5 million in 2024 (Table 1; Figure 1). Thanks to global initiatives aimed at expanding healthcare coverage. However, as access improved, the focus shifted to the quality of care provided, revealing that poor quality care was becoming a leading cause of preventable deaths. The data shows that while the number of deaths due to lack of access decreased from 5 million in 2015 to a projected 3.5 million in 2024, deaths from poor-quality care increased from 5 million to 6 million in the same period (Table 1; Figure 1). This trend highlights the growing importance of healthcare quality as a determinant of health outcomes. Even as more people gain access to healthcare, ensuring that their care is effective, safe, and of high quality is crucial in reducing global mortality rates.

**TABLE 1:** Mortalities due to lack of access to care and poor-quality care: a ten 10-year global trend analysis (2015–2024).

Year	Mortality due to lack of access to healthcare	Mortality due to poor quality care
2015	- Estimated Deaths: 5 million annually (globally)	- Estimated Deaths: 5 million annually (globally)
	- Significant deaths in LMICs due to barriers like financial constraints and geographic isolation	- High mortality due to medical errors, misdiagnoses, and inadequate treatment
	Source: [2]	Source: [1]
2016	- Estimated Deaths: 4.8 million annually (globally)	- Estimated Deaths: 5.2 million annually (globally)
	- Continued reduction due to global health initiatives	- Rising deaths from inadequate healthcare quality
	Source: [6]	Source: [6]
2017	- Estimated Deaths: 4.6 million annually (globally)	- Estimated Deaths: 5.4 million annually (globally)
	- Further improvements in healthcare access	- Increasing mortality from poor quality care
	Source: [6]	Source: [6]
2018	- Estimated Deaths: 4.5 million annually (globally)	- Estimated Deaths: 5.5 million annually (globally)
	- Persistent issues in rural and remote areas	- Ongoing challenges in delivering consistent and safe care
	Source: [21]	Source: [6]
2019	- Estimated Deaths: 4.3 million annually (globally)	- Estimated Deaths: 5.6 million annually (globally)
	- Significant impact of global health efforts to improve access	- Growing recognition of the need for quality care
	Source: [6]	Source: [6]
2020	- Estimated Deaths: 4 million annually (globally)	- Estimated Deaths: 5.7 million annually (globally)
	- COVID-19 pandemic caused a temporary increase in mortality	- The pandemic exacerbated gaps in healthcare quality
	Source: [6]	Source: [6]
2021	- Estimated Deaths: 3.9 million annually (globally)	- Estimated Deaths: 5.8 million annually (globally)
	- Healthcare systems began recovering post-pandemic	- Continued strain on healthcare quality due to pandemic aftereffects
	Source: [22]	Source: [6]
2022	- Estimated Deaths: 3.7 million annually (globally)	- Estimated Deaths: 5.9 million annually (globally)
	- Renewed efforts to improve healthcare access	- Persistent challenges in delivering high-quality care
	Source: [22]	Source: [6]
2023	- Estimated Deaths: 3.6 million annually (globally)	- Estimated Deaths: 6 million annually (globally)
	- Continued reduction, but disparities remain	- Ongoing issues with healthcare safety and effectiveness
	Source: [22]	Source: [6]
2024 (Projected)	- Estimated Deaths: 3.5 million annually (globally)	- Estimated Deaths: 6 million annually (globally)
	- Persistent challenges in reaching the most vulnerable populations	- Quality of care remains a critical issue, especially in LMICs
	Source: [22]	Source: [6]

## Discussion

The assertion that the risk of accessing healthcare has become far greater than the lack of access to healthcare raises essential questions about balancing efforts to improve access with the need to ensure quality and safety in healthcare delivery. While increasing access to healthcare remains a critical goal, it is equally important to address the systemic issues contributing to the risks of harm in healthcare settings [6, 23]. One approach to achieving this balance is to strengthen healthcare systems by investing in quality improvement initiatives, enhancing healthcare worker training, and implementing robust infection control measures. Additionally, there is a need for greater regulation and oversight of healthcare practices, particularly in LMICs, to ensure patients receive safe and effective care [21].

### The Role of Health Policy and Governance

Health policy and governance play a crucial role in mitigating the risks associated with healthcare access. Governments and health authorities must prioritize patient safety and quality of care in their healthcare policies and programs. This includes establishing national frameworks for patient safety, setting standards for healthcare delivery, and ensuring accountability in healthcare systems [24]. In addition to policy efforts at the national level, international organizations such as the WHO can provide guidance and support to countries in developing and implementing patient safety strategies. Collaboration between governments, healthcare providers, and civil society organizations is essential to create a healthcare environment where the risks of harm are minimized, and the benefits of care are maximized [4].

### Ethical Implications and Patient Autonomy

The risks associated with accessing healthcare also have ethical implications, particularly concerning patient autonomy. Patients have the right to make informed decisions about their healthcare, including accepting or declining treatment based on understanding the risks and benefits. However, in many cases, patients may not have access to the information or support they need to make these decisions, particularly in resource-limited settings [25]. Healthcare providers have an ethical responsibility to ensure that patients are fully informed about the potential risks of treatment and to involve them in the decision-making process. This includes providing clear and accurate information about the likelihood of adverse events, the availability of alternative treatments, and the potential outcomes of not receiving care [21, 23, 26]. By respecting patient autonomy and promoting shared decision-making, healthcare providers can help mitigate the risks of accessing healthcare.

### Addressing the Root Causes

Addressing the root causes of these risks is essential to reduce the risks associated with accessing healthcare. This includes tackling the underlying social, economic, and political factors contributing to healthcare system weaknesses and patient vulnerability. For example, addressing poverty, improving education, and strengthening health infrastructure can help create a more resilient healthcare system better equipped to provide safe and effective care [23, 27, 28]. Moreover, efforts to combat AMR, improve infection control practices, and enhance healthcare worker training are critical to reducing the risks of harm in healthcare settings. By addressing these root causes, policymakers and healthcare providers can work towards a healthcare system where the risks of accessing care are minimized, and the benefits of treatment are maximized [11].

### The Future of Healthcare Access and Safety

Looking to the future, the risks associated with healthcare access must be a central consideration in the ongoing efforts to improve global health. As healthcare systems continue to evolve, there will be a need for ongoing monitoring and evaluation of patient safety and quality of care. This will require developing new tools and methodologies for assessing healthcare risks and integrating patient safety into broader health system strengthening efforts [4]. In addition, there is a need for more research and innovation in healthcare delivery, particularly in LMICs, where the risks of harm are often highest. This includes exploring new models of care that prioritize patient safety and developing technologies and interventions that can reduce the risks associated with healthcare access [21, 23, 26].

The assertion that the risk of accessing healthcare is becoming far greater than the lack of access to healthcare highlights the complex and multifaceted nature of healthcare delivery in the modern world. While access to healthcare remains a fundamental goal, ensuring that the care provided is safe, effective, and high-quality is equally essential. The risks associated with healthcare access, including patient safety concerns, healthcare-associated infections, and antimicrobial resistance, must be addressed through concerted efforts at the policy, governance, and practice levels. Ultimately, the goal should be to create a healthcare system where patients can access the care they need without fear of harm and where the benefits of treatment far outweigh the risks. This will require a continued commitment to improving healthcare quality, strengthening healthcare systems, and addressing the root causes of healthcare risks.
